# Peer Review for “Checklist Approach to Developing and Implementing AI in Clinical Settings: Instrument Development Study”

**DOI:** 10.2196/69595

**Published:** 2025-02-20

**Authors:** Anonymous

**Keywords:** artificial intelligence, machine learning, algorithm, model, analytics, AI deployment, human-AI interaction, AI integration, checklist, clinical workflow, clinical setting, literature review

*This is the peer-review report for “Checklist Approach to Developing and Implementing AI in Clinical Settings: Instrument Development Study.”*

## Round 1 Review

This paper [1] introduces the Clinical Artificial Intelligence (AI) Sociotechnical Framework (CASoF), a checklist developed through a literature synthesis and refined by a modified Delphi study. It aims to guide the development and implementation of AI in clinical settings, focusing on the integration of both technological performance and sociotechnical factors. The framework addresses gaps in existing frameworks by emphasizing not only technical specifications but also the broader sociotechnical dynamics essential for successful AI deployment in health care.

New approaches to reporting AI in clinical settings are crucial as AI becomes more integrated into clinical practice. However, the paper needs to address the “black box” dilemma more thoroughly. This refers to the opaque nature of AI algorithms, where the decision-making process is not easily interpretable by clinicians, leading to trust and transparency issues. Additionally, while the CASoF checklist is a valuable tool, it would benefit from a more detailed comparison to established frameworks like TRIPOD (Transparent Reporting of a Multivariable Prediction Model for individual Prognosis or Diagnosis), which has been widely used in developing and validating clinical prediction models. Discussing how the CASoF complements or improves upon TRIPOD would strengthen the paper’s contributions.

I suggest adding a paragraph discussing the potential roles of AI when integrated into hospital electronic health record (EHR) systems. AI could be used for the development of advanced diagnostic and prognostic tools by analyzing real-time patient data. Integration with EHRs could enhance decision-making, providing predictive analytics at the point of care and improving patient outcomes. This would help explore the broader clinical impact of AI beyond just technical integration, addressing its potential for continuous learning and optimization in health care settings.

## Abbreviations

AI: artificial intelligence
CASoF: Clinical Artificial Intelligence Sociotechnical Framework
EHR: electronic health record
TRIPOD: Transparent Reporting of a Multivariable Prediction Model for individual Prognosis or Diagnosis
